# A Unique Case of Testicular Compromise in a Patient with Ovotesticular Disorder of Sexual Development and a Solitary Testicle

**DOI:** 10.1155/2017/8527071

**Published:** 2017-10-08

**Authors:** Ethan Vargo, Lillianne Stanitsas, Mark Memo

**Affiliations:** ^1^Ohio University Heritage College of Osteopathic Medicine, Athens, OH, USA; ^2^Department of Surgery, St. Elizabeth Youngstown Hospital, Youngstown, OH, USA; ^3^N.E.O. Urology Associates, Youngstown, OH, USA

## Abstract

Ovotesticular disorder of sexual development (OT-DSD), previously true hermaphroditism, is a condition in which one or both gonads contain testicular and ovarian tissue. A 23-year-old OT-DSD male patient presented with continuous pain in his right testicle which had been previously intermittent over the past five days. The patient had a prior history of left ovotestis removal with prosthesis placement, a right undescended testicle with aberrant anatomy, and hypospadias repair, all of which were corrected shortly after birth. A lack of blood flow to the testicle on Doppler ultrasound warranted immediate surgical intervention. Intraoperatively, an aberrant tunica vaginalis space with a compressive hematoma secondary to epididymal abscess rupture was identified as the causation for testicular compromise. Return of vascular flow to the testicle was confirmed with intraoperative Doppler after hematoma and epididymis excision, and the testicle was left in situ. It is imperative to consider epididymal etiologies with acute testicular pain, especially in a patient with a medical history that carries an increased risk for gonadal anomalies.

## 1. Introduction

It is widely suspected that the term “hermaphrodite” first appeared in ancient Greek mythology describing Hermaphroditus, the offspring of Hermes and Aphrodite, who possessed both female and male physical characteristics [1]. Today, the umbrella terminology, disorders of sex development (DSD), has largely replaced terms like intersex and hermaphroditism and is generally used to describe someone with ambiguous genitalia or mosaicism of the gonads [2].

Ovotesticular disorder of sexual development (OT-DSD), representing five percent of DSD, is a condition that involves the presence of an ovotestis [3]. The diagnosis of an ovotestis is made histologically, requiring the presence of both seminiferous cords and ovarian follicles with oocytes [4]. The ovotestis commonly identified in OT-DSD patients increases their risk for acquiring adverse gonadal and genitourinary pathologies. Gonadoblastomas, hypospadias, cryptorchidism, and aberrant anatomy are just a few genitourinary anomalies that have been reported in patients with OT-DSD [5, 6]. We report the case of a 23-year-old OT-DSD male with aberrant gonadal anatomy who presented with acute right testicular pain and a unique clinical picture.

## 2. Case Study

**Figure 1:**
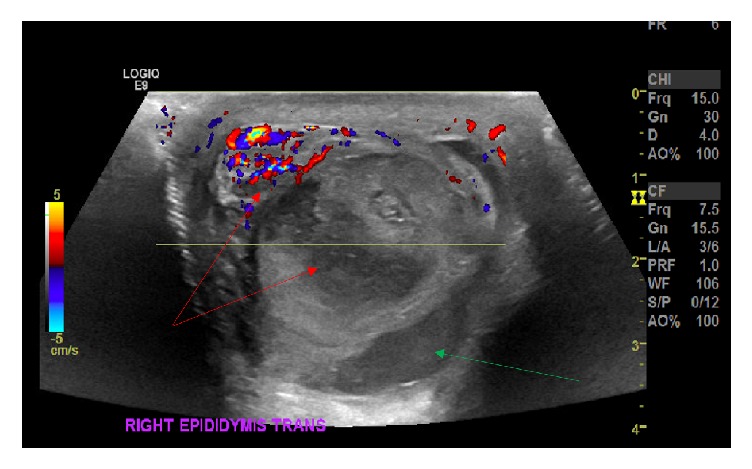
Doppler ultrasound performed upon patient presentation demonstrating heterogeneous echotexture in both the testicle and the epididymis signifying ischemia and inflammation in the testicle and necrosis in the epididymis (red arrows). Only peripheral blood flow to the testicle is present while blood flow to the epididymis is maintained. Additionally, a significant hematoma is visualized on the anterior aspect of the testicle (green arrow).

A 23-year-old male patient presented to the Emergency Department with intermittent testicular pain of five days' duration in his solitary right testicle. Upon presentation, the patient admitted to increasing severity of the pain in his right testicle over the course of the previous few hours. The patient denied any recent history of trauma, injury, prior episodes of pain, or radiation of the pain. The patient and a family member, along with the patient's pediatric urologist, confirmed that he had been diagnosed with OT-DSD at birth and had previously undergone left ovotestis removal with subsequent prosthesis placement, an orchiopexy for an undescended right testicle, and correction of hypospadias, all taking place shortly after birth. On physical examination, the patient appeared phenotypically male. The patient's exam was remarkable for a fixed and indurated testicle on the right side. Suspecting either testicular torsion, possible mass, or testicular abscess, an ultrasound with Doppler was performed. The ultrasound demonstrated prominent right epididymal vascularity and marked abnormality of the right testicle along with heterogeneous echotexture with solid sonographic characteristics throughout the right scrotal sac (Figure 1). Additionally, Doppler ultrasound demonstrated only peripheral blood flow and a lack of central flow to the testicle, while blood flow was preserved within the epididymis (Figure 1). With these findings, the patient was taken to surgery for right testicular exploration within one hour and thirty minutes hours of his presentation.

**Figure 2:**
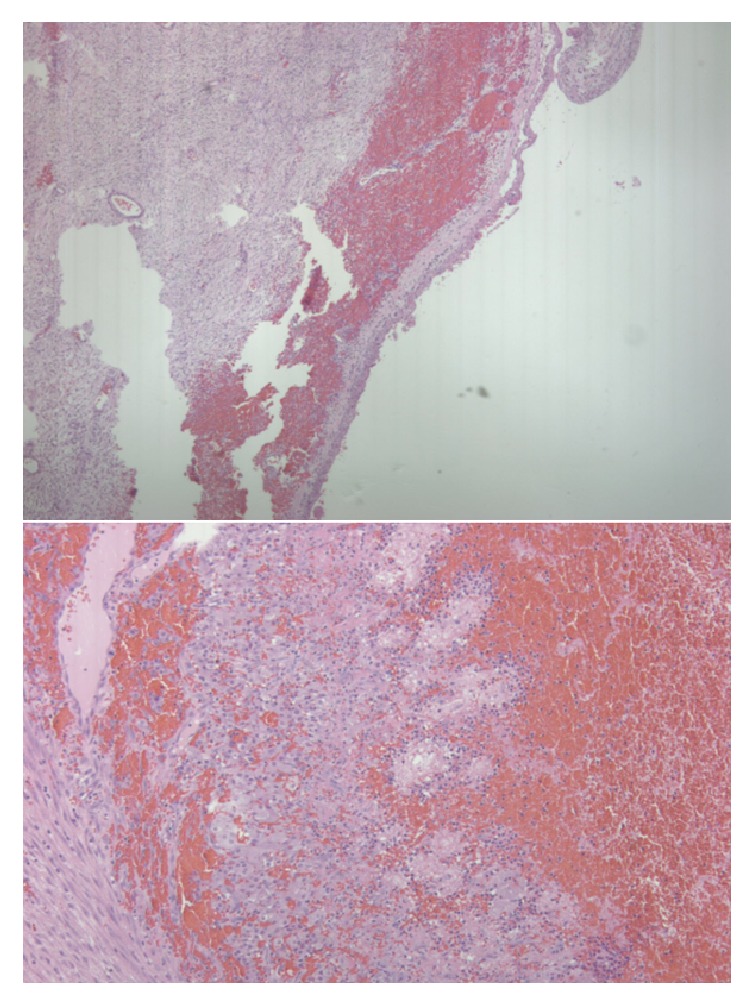
Histology of specimen collected intraoperatively demonstrating coagulative necrosis of epididymal tissue with surrounding hematoma.

**Figure 3:**
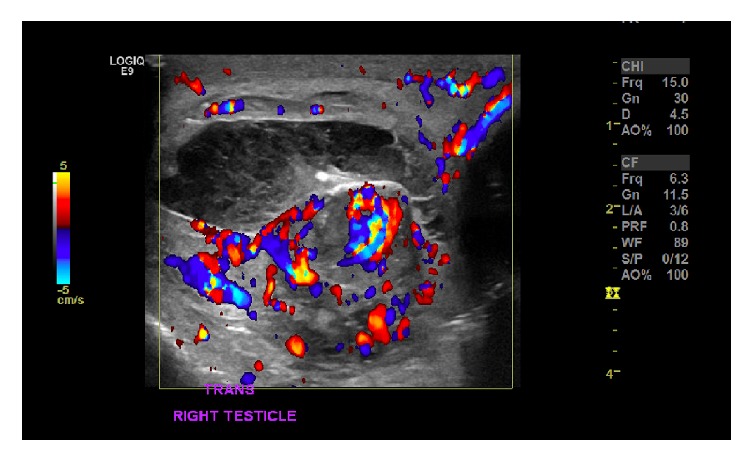
Doppler ultrasound on postoperative day one of the right testicle demonstrating robust return of vascular flow to the testicle following necrotic epididymis and hematoma excision.

Due to the risk of finding a testicular neoplasm, a right inguinal incision was used to explore the right testicle. Intraoperatively, a necrotic epididymis as a result of epididymal abscess rupture had led to hematoma formation which was compressing the testicle and compromising vascular flow. The patient had aberrant anatomy with a small confined space from the tunica vaginalis. No degree of torsion of the testicle was appreciated. The hematoma was decompressed, the necrotic epididymis was excised, and an intraoperative Doppler was performed in order to evaluate vascular flow. Following decompression of the hematoma, intraoperative Doppler confirmed the return of vascular flow to the patient's solitary right testicle. The testicle was left in situ, as no other anatomic abnormalities were observed. The hematoma and epididymis were sent for pathology, and histology confirmed that the specimen was comprised of necrotic epididymis and surrounding hematoma (Figure 2). The patient was admitted for one day and received a follow-up ultrasound on postoperative day one which exhibited robust return of vascular flow to the right testicle, and he was discharged home (Figure 3).

## 3. Discussion

Our patient's pertinent history and curious presentation along with his physical exam and ultrasound findings allowed our team to focus on weighing torsion versus malignancy versus abscess. Acute testicular pain is associated with numerous differential diagnoses, and while certain physical exam findings may suggest one diagnosis over another, the final and most definitive step in the physical exam for acute testicular pain is ultimately surgical exploration [7]. Surgical exploration was especially warranted in our patient, as a preoperative Doppler ultrasound demonstrated minimal blood flow to the testicle, solid sonographic characteristics concerning for fluid accumulation, and heterogeneous echotexture in the epididymis and testicle suggestive of inflammation.

Our physical exam findings of a fixed, indurated, and exquisitely tender testicle were validated with our intraoperative findings. Due to an episode of epididymitis, increased blood flow to the epididymis had resulted in excessive inflammation and abscess formation. While blood flow to the epididymis was maintained, the epididymal abscess likely ruptured and resulted in necrosis and hematoma formation around the epididymis, subsequently resulting in compression of the testicle. This hematoma ultimately accounted for the induration that was palpated on physical exam and was responsible for the patient's pain due to testicular ischemia. The patient's enhanced pain over the course of a few hours prior to presentation likely occurred secondary to significant compression of testicular vasculature due to hematoma growth. While we were able to salvage the testicle with evacuation of the hematoma, the epididymis appeared necrotic and it was excised.

While epididymitis in adult males under 35 years of age is commonly due to sexual activity, other noninfectious causes like trauma, vasculitis, and autoimmune disease can also be responsible [8]. Though these previously mentioned causes were ruled out in our patient, we argue that this case of hemorrhagic epididymitis was due to our patient's aberrant genitourinary anatomy secondary to his past medical history of OT-DSD. As previously established, higher rates of cryptorchidism have been observed in patients with DSD, particularly OT-DSD [9, 10]. Cryptorchidism is the lack of descent of the testicle and associated layers of the abdominal peritoneum into the scrotum during embryonic development, explaining the underdeveloped tunica vaginalis observed in our patient. Additionally, epididymal abnormalities have been shown to occur more frequently in patients with cryptorchidism, with rates as high as 72% [11]. While no macroscopic abnormalities of our patient's epididymis or its vasculature were appreciated intraoperatively, it is certainly plausible that aberrant development of this patient's solitary epididymis could have contributed to his condition. Therefore, our patient's original diagnosis of OT-DSD increased his risk for cryptorchidism which subsequently increased his risk for developing an epididymal anatomic abnormality, ultimately leading to this acute presentation.

Another pertinent aspect of this case involved preserving this patient's solitary testicle. Had the necessary surgical measures not been swiftly taken, this patient could have undergone a second orchiectomy, rendering him anorchid. Additionally, the patient avoided potential subjection to social anxieties related to possessing two testicular prostheses.

In conclusion, this case serves to educate and inform the practitioner of a more complete list of the causes of acute testicular pain and to carefully consider the patient's medical history when arriving at a diagnosis. Certainly, there is a role for the traditional physical exam when evaluating a patient initially; however, the final step in a urologist's evaluation of acute scrotal pain without an identifiable cause is surgical exploration. Additionally, this case highlights the importance of a surgeon's preoperative and intraoperative decision-making on a patient's quality of life.
